# Ultrafast Self‐Driven WSe_2_ Photodetectors with Bottom Schottky Contacts

**DOI:** 10.1002/advs.202510373

**Published:** 2025-08-04

**Authors:** Jian Li, Zhihao Wang, Jialing Jian, Zhengjin Weng, Qianqian Wu, Xingyu Zhou, Liangliang Lin, Xiaofeng Gu, Peng Xiao, Haiyan Nan, Shaoqing Xiao

**Affiliations:** ^1^ Engineering Research Center of IoT Technology Applications (Ministry of Education) School of Integrated Circuits Jiangnan University Wuxi 214122 China; ^2^ School of Chemical and Material Engineering Jiangnan University Wuxi 214122 China; ^3^ Université de Bordeaux, LOMA, CNRS UMR 5798 Talence F‐33400 France

**Keywords:** 2D Photodetector, asymmetric electrodes, bottom electrode, Se buffer layer, ultrafast response

## Abstract

Conventional top‐contact two dimensional (2D) Schottky photodetectors suffer from light shadowing and contact damage, leading to Fermi‐level pinning and performance degradation. This work overcomes these limitations by designing a bottom‐electrode Schottky photodetector (BE‐Schottky PD) based on a Cr/WSe_2_/Au heterostructure. The key innovation involves fabricating the bottom Schottky Cr electrode into pre‐etched SiO_2_ substrate trenches, making it flush with the surface. This unique geometry eliminates optical shadowing to maximize light absorption, and enables a high‐quality van der Waals Cr/WSe_2_ interface, mitigating Fermi‐level pinning. Consequently, the device exhibits an outstanding rectification ratio of 1.07 × 10^4^ and an ideality factor of 1.11 due to the strong built‐in electric field. It demonstrates excellent self‐powered operation within the visible spectrum. Under 532 nm laser illumination and zero bias, it achieves rapid photoresponse with a fall time of 3.8 µs. This work, utilizing industry‐compatible metals and a simple process, realizes a high‐performance photodetector, highlighting the significant potential of 2D materials for efficient, low‐power, and ultrasensitive optoelectronics.

## Graphical abstract

A Schottky photodetector is designed with embedded bottom electrodes, enabling the formation of a high‐quality van der Waals interface and significantly enhancing the device's photoresponsivity and response speed. This work demonstrates a high‐performance Schottky photodetector realized using industry‐compatible metal materials and a simplified fabrication process, highlighting the tremendous potential of 2D materials in the development of efficient, low‐power, and ultrasensitive optoelectronic devices.

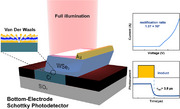

## 1 Introduction

In recent years, two dimensional (2D) materials have garnered significant attention in the fields of condensed matter physics and nanoelectronics due to their atomic‐scale thickness, high specific surface area, and tunable electronic properties.^[^ ^1^ ^]^ To further harness the potential of 2D materials in device applications, researchers have intensively studied heterostructures^[^ ^2^ ^]^ formed by stacking different 2D materials in vertical or lateral configurations. These heterojunctions enable a range of functionalities‐including carrier separation, rectification, and photovoltaic conversion‐on a single platform and have been successfully applied in transistors, photodetectors, light‐emitting diodes, and more.^[^ ^3^ ^]^ However, the construction of heterojunctions typically relies on complex interlayer transfer techniques, which significantly increase fabrication complexity and often introduce contamination or voids at the interfaces.^[^ ^4^ ^]^ These issues can lead to band discontinuities, enhanced interface scattering, and ultimately compromise device stability and reproducibility.^[^ ^5^ ^]^

In contrast, homojunctions, which involve creating electrically distinct regions within the same 2D material, offer inherent advantages such as perfect lattice matching, defect‐free interfaces, and efficient carrier transport.^[^ ^6^ ^]^ Particularly for atomically thin materials, their electronic structures are highly sensitive to external perturbations, allowing for the possibility of engineering functional regions within a single flake through local modulation.^[^ ^7^ ^]^ This enables the realization of functionalities typically attributed to conventional heterojunctions.^[^ ^8^ ^]^ However, no doping method for 2D materials currently offers the ideal combination of high spatial resolution, long‐term stability, and low fabrication complexity, which poses a significant challenge for large‐scale integration of 2D materials into electronic and optoelectronic systems.

Against this backdrop, asymmetric metal‐semiconductor Schottky contact^[^ ^9^ ^]^ structures have emerged as a promising alternative. By selecting metals with distinctly different work functions for contact with a 2D material, an internal built‐in electric field can be naturally formed within the material, effectively inducing an intrinsic junction. This approach eliminates the need for complex transfer or doping processes and allows for tunable carrier type and distribution by simply adjusting the electrode materials and layout.^[^ ^10^ ^]^ It offers clear advantages in terms of structural simplicity, fabrication controllability, and device stability.^[^ ^11^ ^]^ However, in such Schottky contact photodetectors, electrodes are typically deposited on top of the 2D material, creating a light shadowing effect that impedes further improvement in light absorption and photoresponsivity. Additionally, conventional top‐contact approaches such as thermal/electron‐beam evaporation involve the bombardment of the contact area with hot metal atoms or clusters. This process causes significant damage through kinetic energy transfer or chemical reactions between metal atoms and the 2D semiconductor, ultimately leading to strong Fermi‐level pinning at the metal‐semiconductor interface and consequent degradation of device performance.

In this study, we designed and fabricated a bottom‐electrode Schottky photodetector (BE‐Schottky PD) based on a 2D Cr/WSe_2_/Au structure. The bottom Schottky Cr electrode, which is flush with the SiO_2_ surface, was deposited into pre‐etched trenches on the SiO_2_ substrate. A WSe_2_ flake was then mechanically transferred onto the substrate surface, establishing van der Waals contacts with the embedded electrodes. The bottom electrode configuration avoids the shadowing effect, thereby improving light absorption and responsivity, and enhances carrier generation near the depletion region, supporting self‐powered operation. Moreover, the structure forms clean van der Waals contacts, minimizing Fermi level pinning and boosting photoresponse speed. To further optimize the device, the top Ohmic contact was fabricated in a staggered geometry using Se/Au, where the Se buffer layer plays a critical role in passivating interface defects, mitigating Fermi level pinning, and improving adhesion between the 2D material and metal electrode.^[^ ^12^ ^]^ This interface engineering significantly enhances carrier injection efficiency and contributes to improved device performance and long‐term stability.

We systematically compared field‐effect transistor (FET) devices based on Cr‐WSe_2_‐Cr and Au‐WSe_2_‐Au configurations. The Schottky barrier height (SBH) extracted from thermionic emission theory was approximately 101 meV. Furthermore, we performed a comparative study between top‐contact and bottom‐contact device architectures, demonstrating that the bottom‐contacted WSe_2_ Schottky photodetector exhibits superior electrical and optoelectronic performance. Thanks to the strong built‐in electric field at the Cr/WSe_2_ interface, the device delivers outstanding performance metrics, including an ultra‐low dark current in the pA range, excellent rectification behavior (rectification ratio of 1.07 × 10^4^, ideality factor of 1.11), a fast fall time of 3.8 µs under 532 nm illumination, and a responsivity of 360 mA W^−1^ with a detectivity of 1.07 × 10^12^ Jones at 447 nm. This work presents a simple yet high‐performance and complementary metal‐oxide‐semiconductor (CMOS)‐compatible 2D photodetector design, offering an effective solution for next‐generation high‐speed, low‐power optoelectronic communication and AI‐driven sensory applications.

## 2 Results and Discussion

**Figure 1:**
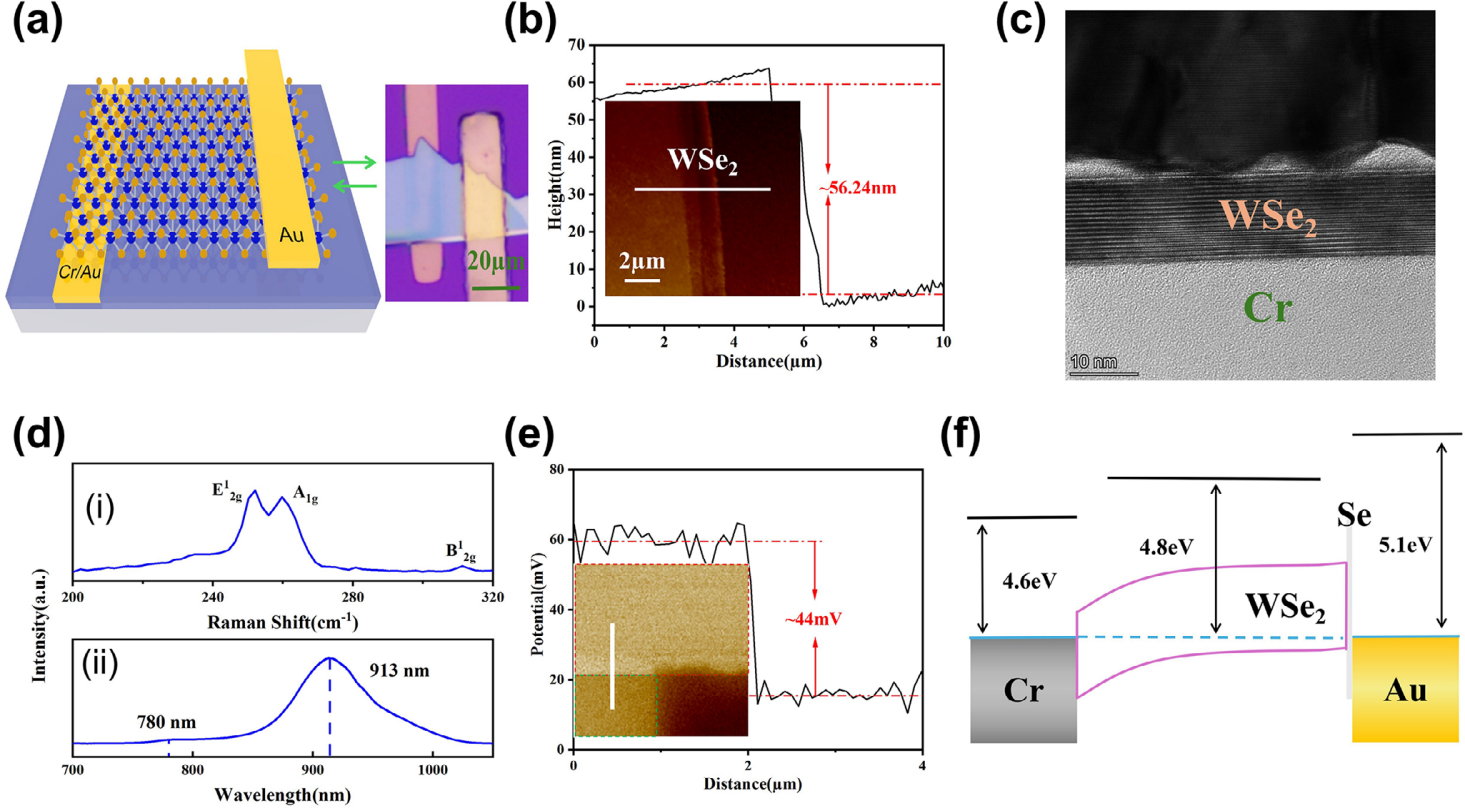
Conceptual Illustration and Characterization of the Cr/WSe_2_/Au BE‐Schottky PD. a) The left panel shows the diagram of BE‐Schottky PD, while the right panel presents the optical microscope image of the device. b) Atomic Force Microscopy (AFM) image of the device (inset). The height profile along the white line indicates that the WSe_2_ flake is 56.24 nm. c) HAADF‐STEM image of the embedded Cr electrode in contact with a thin WSe_2_ layer. Here the thin WSe_2_ layer instead of the pristine thick WSe_2_ layer was chosen for the purpose of HAADF‐STEM characterization. d) (i) Raman and (ii) PL spectra of multilayer WSe_2_. e) KPFM image of the device (inset). The potential profile along the white line shows a potential difference of 44 mV between the WSe_2_ and the Au electrode. f) Energy band alignment diagram of WSe_2_ interfaced with metal contacts.

**Figure** **1**a illustrates the schematic diagram and the optical microscope image of the Cr‐WSe_2_‐Au Schottky photodetector. This device employs embedded bottom electrodes and utilizes a selenium (Se) buffer layer to overcome the Fermi level pinning effect commonly associated with conventional thermally evaporated electrodes, thereby achieving outstanding optoelectronic performance.^[^ ^4^ ^]^ Details of the fabrication process are provided in Figure S1 (Supporting Information). To determine the thickness of the WSe_2_ flake, atomic force microscopy (AFM) was performed, and the results are presented in Figure 1b, revealing a flake thickness of ≈56.24 nm. Figure 1c shows a high‐angle annular dark‐field scanning transmission electron microscopy (HAADF‐STEM) image of the interface between the embedded Cr electrode and WSe_2_ (a thin WSe_2_ layer was used for HAADF‐STEM characterization). A clean and well‐defined interface can be clearly observed. Figure 1d(i) shows the Raman spectrum of WSe_2_ under 532 nm laser excitation, where three characteristic peaks corresponding to the *E^1^*_2g_, *A_1g_*, and *B^1^_2g_* modes are observed at 252.1 cm^−1^, 259.8 cm^−1^, and 311.2 cm^−1^. It is evident that the WSe_2_ sample consists of multilayer WSe_2_.^[^ ^13^ ^]^ Figure 1d(ii) presents the photoluminescence (PL) spectrum of the WSe_2_ film, where two emission peaks are observed: one at 780 nm corresponding to the direct bandgap and another at 913.5 nm corresponding to the indirect bandgap. Since the WSe_2_ sample is multilayered, the bandgap is determined based on the indirect transition. The bandgap energy can be calculated using the following Equation (1):

(1) $$E=\frac{hc}{\lambda }\;$$

where *E* is the photon energy, h is Planck's constant, c is the speed of light, and *λ* is the wavelength. Based on our calculations, the PL peak energy is 1.36 eV. Since the PL peak energy is approximately equal to the bandgap of WSe_2,_^[^ ^14^ ^]^ the bandgap of WSe_2_ is therefore estimated to be ≈ 1.36 eV. To evaluate the potential difference between WSe_2_ and the Au electrode, Kelvin probe force microscopy (KPFM) measurements were performed, as shown in Figure 1e, the results reveal a potential difference of ≈44 mV. The Fermi level of WSe_2_ is approximately 4.8 eV,^[^ ^3^ ^]^ the work function of Au is 5.1 eV.^[^ ^15^ ^]^ Experimental measurements reveal that the potential difference between WSe_2_ and Au is only 44 meV, indicating that the SBH is significantly lower than theoretically expected. This discrepancy is primarily attributed to the introduction of a Se buffer layer at the interface, which effectively improves the interfacial quality between the metal and WSe_2_. The buffer layer reduces the density of interface defect states and suppresses Fermi level pinning, thereby minimizing band bending at the contact. The energy band diagram of the WSe_2_ Schottky photodetector can be deduced, as shown in Figure 1f.

**Figure 2:**
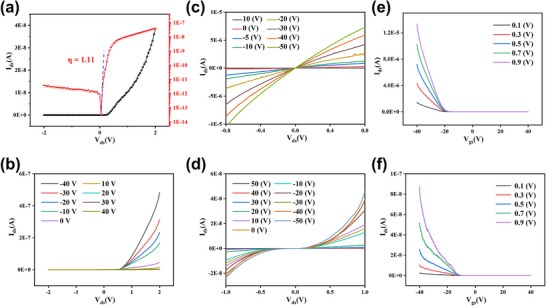
Electrical Characteristics of the WSe_2_ Device with Cr‐Au Contacts. a) I_ds_‐V_ds_ characteristics of the WSe_2_ Schottky diode with Cr‐Au contacts, shown on both linear and logarithmic scales. The extracted ideality factor of the Schottky diode is 1.11. b) I_ds_‐V_ds_ curves of the WSe_2_ Schottky diode under gate voltages ranging from −40 to 40 V. c) I_ds_‐V_ds_ curves of the device with Au–Au contacts under gate voltages ranging from −50 to 10 V. d) I_ds_‐V_ds_ curves of the device with Cr–Cr contacts under gate voltages ranging from −50 to 50 V. e) I_ds_‐V_gs_ curves of the Au–Au contact device measured at various drain voltages. f) I_ds_‐V_gs_ curves of the Cr–Cr contact device measured at various drain voltages.

**Figure** **2**a shows the I_ds_‐V_ds_ characteristics of the WSe_2_ Schottky diode with a bottom electrode configuration. The device exhibits typical Schottky diode behavior, with forward conduction and reverse blocking characteristics, achieving a high rectification ratio of 1.07 × 10^4^. To evaluate the ideality factor (*η*) in the forward bias region, the Shockley diode equation can be simplified as follows:

(2) $$\eta \;=\frac{q}{k_{B}T\frac{d\left(\ln I\right)}{dV}}$$

where q is the elementary charge (1.6 × 10^−19^ C), *k_B_* is the Boltzmann constant (1.38 × 10^−23^ J K^−1^), and *T* is the Kelvi temperature.^[^ ^16^ ^]^ The calculated ideality factor is 1.11, indicating a high‐quality Schottky diode, as values closer to 1 represent more ideal diode behavior. Figure 2b demonstrates that the device maintains excellent rectifying behavior under various gate voltages. Figure 2c,d displays the I_ds_‐V_ds_ characteristics of devices with Au–Au and Cr–Cr contacts, respectively, under different gate voltages. The output curves in Figure 2c exhibit a clear linear behavior. In contrast, the output characteristics in Figure S2 (Supporting Information), where Au electrodes were directly deposited, show less linearity compared to the devices with a Se buffer layer.^[^ ^4^, ^12^ ^]^ This indicates that the combination of Se buffer layer deposition followed by annealing to remove it enables the formation of a high‐quality Ohmic contact between Au and WSe_2_. Conversely, Figure 2d shows that Cr–Cr contacts produce nonlinear output characteristics, suggesting the formation of a high‐resistance Schottky contact. This behavior stems from the lower Fermi level of WSe_2_ relative to Cr (but closer to that of Au), which causes hole diffusion from WSe_2_ into Cr and the formation of a built‐in electric field at the WSe_2_/Cr interface. Under this field, electron drift increases until Fermi level alignment is achieved, resulting in a Schottky barrier that impedes carrier transport at the metal‐semiconductor junction. In contrast, the negligible barrier at the Au‐WSe_2_ interface facilitates efficient carrier injection and transport. Figure 2e,f shows the I_ds_‐V_gs_ transfer characteristics of WSe_2_ devices with Au–Au and Cr–Cr contacts, respectively, under different drain biases. Based on the transfer curves shown in Figure 2e,f, the field‐effect mobility (µ_*FE*_) at a 0.9 V drain bias was calculated using the following Equation (3):

(3) $$\mu_{FE}=\frac{L}{W}\;\cdot \frac{\partial V_{D}}{\partial V_{G}}\cdot \frac{1}{C_{OX}V_{D}}$$

where *L* and *W* are the channel length and width, respectively, $\frac{\partial V_{D}}{\partial V_{G}}$ is derived from the slope of the I_ds_‐V_gs_ curve, and *C_ox_* (0.0121 µF cm^−2^) is the capacitance of the 285 nm thick SiO_2_ dielectric. The calculated field‐effect mobility for the Au–Au contact device is 165.3 cm^2^·V^−1^·s^−1^ with an on/off ratio of 10^6^, while the Cr–Cr contact device exhibits a mobility of 8.26 cm^2^·V^−1^·s^−1^ and an on/off ratio of 10^3^. These significant differences in mobility and switching behavior further confirm that Au forms an Ohmic contact with WSe_2_, whereas Cr forms a Schottky barrier.

**Figure 3:**
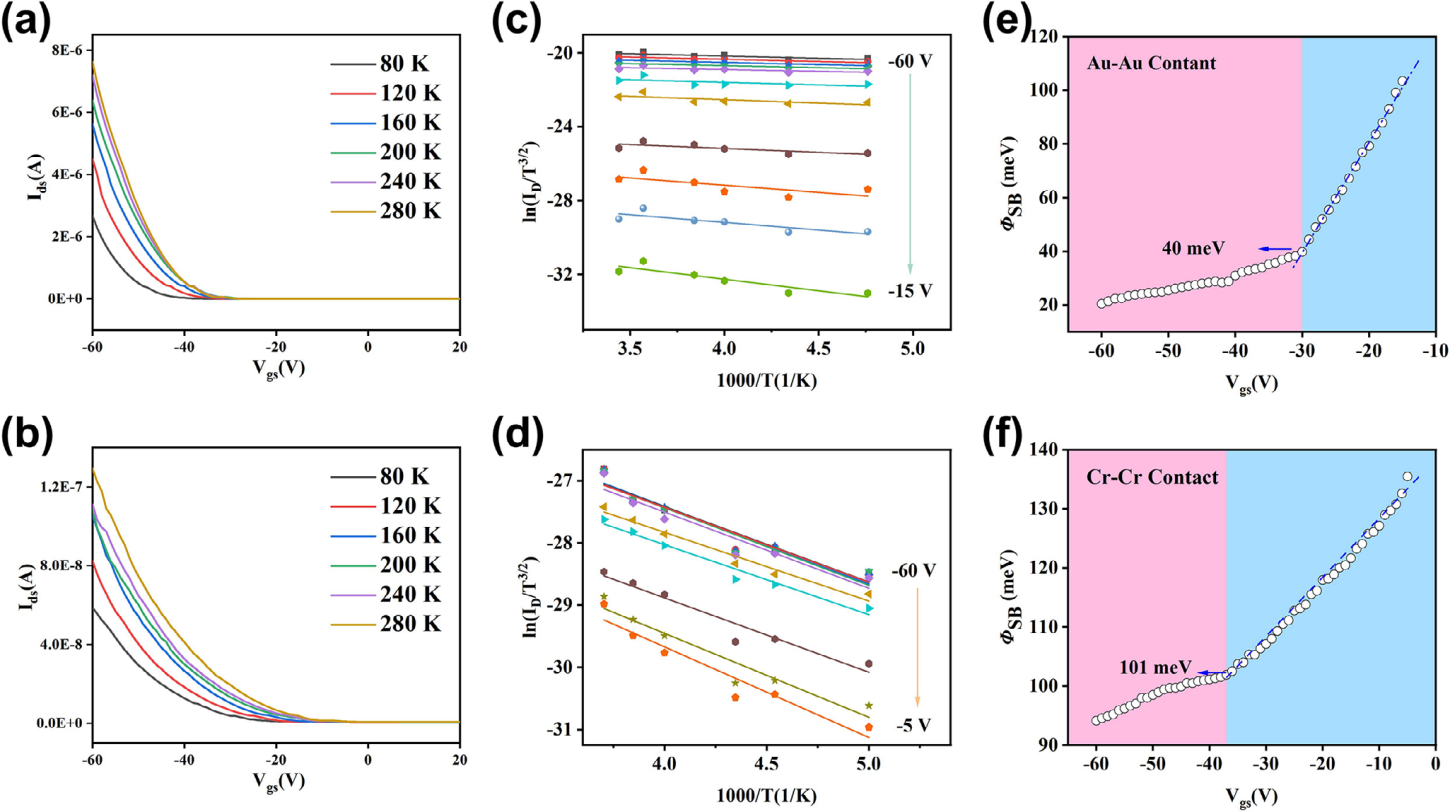
Transfer Characteristics and SBH Analysis of WSe_2_ Devices with Au–Au and Cr–Cr Contacts at Various Temperature. a) Transfer characteristic curves (I_ds_‐V_gs_) of WSe_2_ devices with Au–Au contact electrodes measured at different temperatures. b) Transfer characteristic curves (I_ds_‐V_gs_) of WSe_2_ devices with Cr–Cr contact electrodes measured at different temperatures. c,d) Arrhenius plots for WSe_2_ contacted with (c) Au and (d) Cr under various gate voltages, illustrating the temperature‐dependent transport behavior. e,f) Functional relationship between the effective SBH and V_gs_ for each contact type.

To quantitatively investigate the SBH, we measured the transfer characteristics of WSe_2_ devices with different electrode contacts under low‐temperature conditions. **Figure** **3**a,b displays the temperature‐dependent transfer curves for devices with Au–Au and Cr–Cr contacts, respectively. Based on the thermionic emission theory, the SBH can be calculated using the following Equation (4):^[^ ^17^ ^]^

(4) $$I_{ds}=A_{2D}^{*}\;T^{3/2}\exp\left(-\frac{q\emptyset_{B}}{k_{B}T}\right)\left[1-\exp\left(-\frac{qV}{k_{B}T}\right)\right]$$

where $A_{2D}^{*}$ is the effective 2D Richardson constant, *T* is the absolute temperature, q is the elementary charg, *k_B_* is Boltzmann's constan, ∅_*B*_ is the SBH, and *V* is the drain voltage (set to 1 V in this study). To accurately extract ∅_*B*_, the values of $\ln(\frac{I_{ds}}{T^{3/2}})$ are extracted from transfer curves at various gate voltages, and plotted to construct Arrhenius plots (shown in Figure 3c for Au–Au and Figure 3d for Cr–Cr contacts). By fitting the slopes of these curves, we obtain SBH values corresponding to different V_gs_, as summarized in Figure 3e,f. This analysis establishes the relationship between SBH and gate voltage, revealing how the barrier height modulates with V_gs_ under different contact configurations. The flat‐band voltage (V_FB_) is determined by the intrinsic band alignment between the semiconductor and the metal, as well as the presence of fixed charges at the interface. In this work, we define V_FB_ as the gate voltage (V_gs_) at which the flat‐band condition is achieved, i.e., V_FB_ = V_gs_ when the energy bands of the semiconductor become flat near the contact. Under this condition, carrier transport is primarily governed by thermionic emission over the Schottky barrier. As V_gs_ increases beyond V_FB_, the band bending becomes more pronounced, and thermally assisted tunneling emerges as the dominant conduction mechanism. Experimentally, the extracted SBH values for Au–Au and Cr–Cr contact devices are 40 meV and 101 meV, respectively. This significant contrast further confirms the earlier electrical characterizations, demonstrating that the Cr contact forms a much higher Schottky barrier compared to Au.^[^ ^15b^ ^]^

Figure S3 (Supporting Information) shows the output characteristics of the Au–Au contact device under varying temperature conditions. In Figure S3a (Supporting Information), the I_ds_‐V_gs_ curves at V_gs_ = ‐60 V exhibit nearly ideal linear behavior at low temperatures, indicating that the Au electrodes form excellent Ohmic contacts with WSe_2_. The current‐voltage linearity can be quantified using the nonlinearity factor:

(5) $$N\;=\frac{d^{2}I_{ds}}{dV_{ds}^{2}}/2\frac{dI_{ds}}{dV_{ds}}$$

A smaller *N* value suggests a lower Schottky barrier at the metal‐semiconductor interface.^[^ ^18^ ^]^ As shown in Figure S3b (Supporting Information), the *N* value remains close to zero across all temperature conditions, confirming that the Au‐WSe_2_ interface exhibits a negligible Schottky barrier.

**Figure 4:**
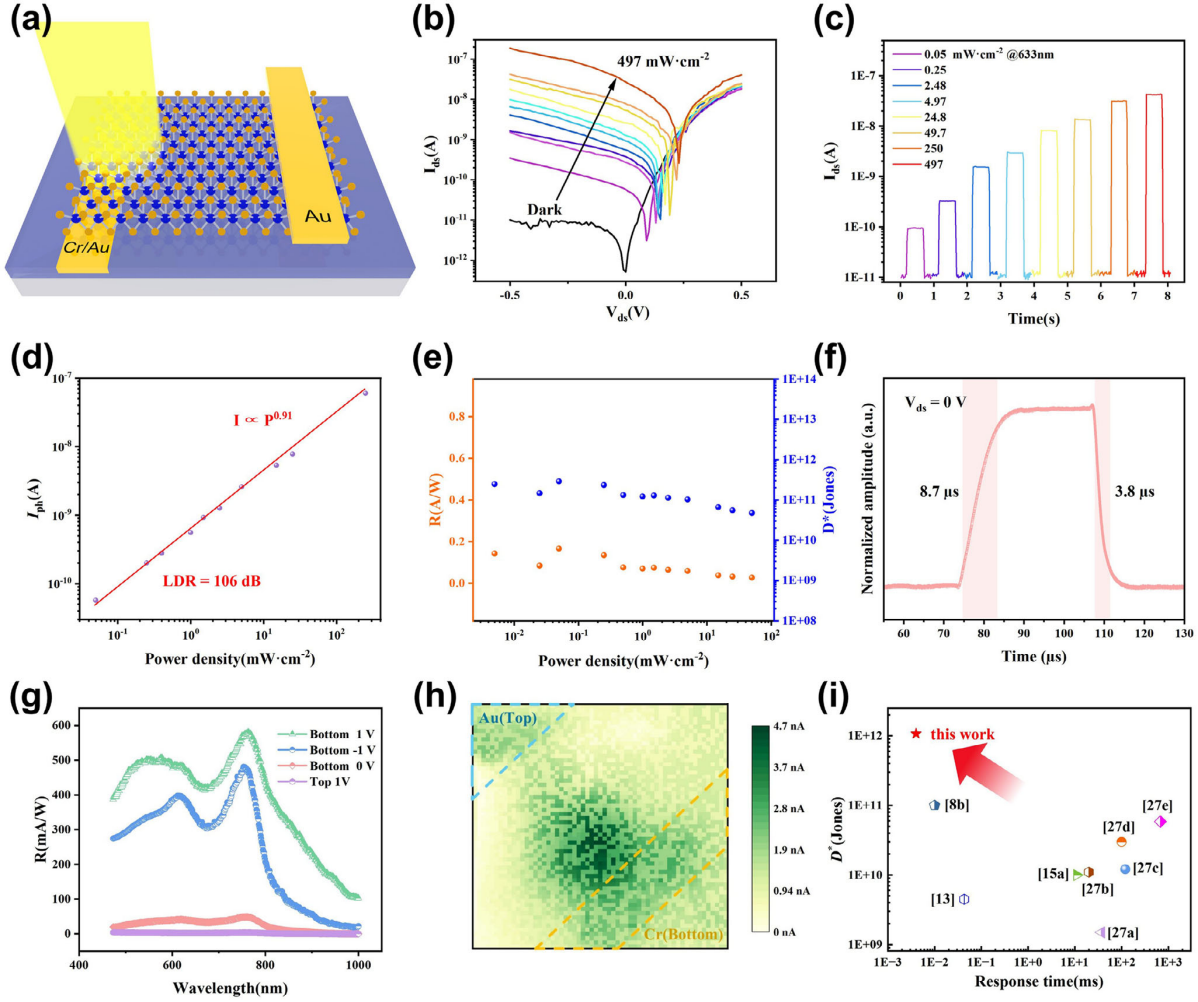
Photodetection Characteristics of the WSe_2_ Schottky Diode a) Schematic diagram of the WSe_2_ Schottky diode photodetector. b) I_ds_‐V_ds_ characteristics of the WSe_2_ Schottky diode under 633 nm laser illumination, with the incident power density ranging from 0.05 to 497 mW·cm^−2^. c) Photocurrent response under varying illumination power densities. d) Photocurrent (I_ph_ = I_light_ – I_dark_) as a function of incident power density extracted from panel (c) at V_ds_ = 0 V. The red line represents a power‐law fit to the experimental data. e) Responsivity (R) and specific detectivity (D*) as a function of illumination power density at V_ds_ = 0 V. f) Response time of the device under 520 nm laser illumination at V_ds_ = 0 V. g) Spectral responsivity of WSe_2_ devices with bottom and top electrodes under different V_ds_ values at constant optical power density. h) SPCM image obtained using a focused 532 nm laser (power = 50 µW). i) Plot of specific detectivity versus response time for intrinsic WSe_2_‐based Schottky photodetectors, compared with reported WSe_2_ Schottky photodiodes in the literature.

Currently, we have successfully fabricated a WSe_2_ Schottky diode that exhibits a rectification ratio as high as 1.07 × 10^4^ and a dark current in the picoampere range under reverse bias. The next section will systematically investigate the optoelectronic characteristics of this bottom‐contact WSe_2_ Schottky diode. **Figure** **4**a illustrates the structure of the WSe_2_ Schottky photodetector. The Cr electrode, acting as the bottom contact, does not obstruct light signal input. Moreover, photogenerated carriers are closer to the depletion region near the bottom electrode, making them easier to separate. The Schottky barrier at the Cr interface effectively blocks dark carriers, while photogenerated carriers are efficiently accelerated. Figure 4b shows the I_ds_‐V_ds_ characteristics of the device under dark conditions and under illumination with a 633 nm wavelength light source, at various light power densities ranging from 0.05 to 497 mW·cm^−2^. The extremely high optical switching ratio arises from the strong Schottky barrier of the device and the low dark current caused by carrier depletion effects.

To evaluate the stability of the device, we remeasured its photoresponse performance after one year. Additionally, we conducted a 14‐h continuous on/off switching test and a 17‐h current stability test under a constant bias at room temperature and atmospheric pressure, as shown in Figure S4 (Supporting Information). Under a 1 V bias, the current gradually decreased during the first 2 h and then stabilized ≈3 × 10^−7^ A. During the 14‐h switching cycle test, the device consistently maintained an on/off ratio of 10^3^, confirming the excellent long‐term stability and reproducibility of the photodiode.

Figure 4c investigates the transient optical response of the device under 0 V bias and 0 V gate voltage with 633 nm laser illumination. The photocurrent increases rapidly and stabilizes with increasing power density, as the number of photogenerated carriers is proportional to the incident photon flux. Typically, the effect of light power on photocurrent can be described by a power‐law relationship, i.e., I_PC_ ∝ P^α^.^[^ ^8^, ^11^ ^]^ For an ideal p‐n junction photodiode, we expect α = 1, meaning the photocurrent is linearly related to the light intensity. However, due to the introduction of intermediate energy states (such as defects, impurities, or structural disorder), which act as traps or recombination centers for carriers, many reported low‐dimensional photodetectors exhibit a sublinear (0 < α < 1) power‐law relationship, referred to as the photoconductive gain effect. As a result, the responsivity of these detectors typically decreases as the light intensity increases, because trap states gradually fill up. By fitting the experimental data (Figure 4d) to the power‐law relationship, we obtained an exponent value close to 1 (0.91), indicating that the photocurrent of this device maintains a good linear relationship over a range of five orders of magnitude in light intensity (from 0.05 to 497 mW·cm^−2^). The linear dynamic range (LDR) was calculated using the Equation (6):

(6) $$LDR=20log_{10}\left(P_{max}/P_{min}\right)$$

The calculated LDR is 106 dB, slightly lower than that of commercial silicon detectors (120 dB) but higher than that of InGaAs detectors (66 dB).^[^ ^19^ ^]^ The LDR is a key metric for applications such as imaging and optical power measurement, directly impacting image reconstruction quality and measurement accuracy. This wide LDR characteristic provides significant advantages for practical applications.^[^ ^20^ ^]^

To further evaluate the photoresponse performance of the device, two key figures of merit were assessed: responsivity (R) and specific detectivity (D*). Figure 4e illustrates the dependence of both R and D* on the incident light intensity. R is defined as the photocurrent generated per unit incident optical power on the effective area of the phototransistor. The specific formula for calculating responsivity is given as follows:^[^ ^9a^ ^]^

(7) $$R=\frac{I_{ph}}{P\times S}\;$$

where *I_ph_* is the photocurrent induced by the incident light, *P* denotes the light intensity, and *S* refers to the active area of illumination. Due to the linear dependence of photocurrent on optical power, R remains nearly constant across the entire range of incident power densities, maintaining a value of ≈180 mA W^−1^. This stability in responsivity is a hallmark of the photovoltaic effect, indicating that photoconductive gain is not the dominant mechanism. Beyond R, D*‐a critical parameter reflecting the device's ability to detect weak optical signals‐was also evaluated. D* is calculated using the Equation (8):^[^ ^11b^ ^]^

(8) $$D^{*}=RA^{1/2}/{\left(2qI_{dark}\right)}^{1/2}$$

where *A* is the effective area of the device, q is the elementary charge, and *I_dark_* is dark current. It should be noted that although the thermal noise and frequency‐dependent 1/*f* noise are neglected in above equation, it is an acceptable simplification especially under a biased and high‐frequency condition,^[^ ^21^ ^]^ so this method is used widely in the literature.^[^ ^22^ ^]^ Based on this formula, the D* values in Figure 4e were derived. Similar to R, D* exhibits minimal variation with light intensity, and under zero‐bias conditions, it reaches as high as 1 × 10^11^ Jones, highlighting the device's excellent low‐light detection capability. Figure S5 (Supporting Information) presents the noise equivalent power (NEP) and external quantum efficiency (EQE) under 633 nm illumination at zero bias. The NEP achieves a minimum of 1.09 × 10^−14^ W·Hz^−1/2^, calculated using NEP = A^1/2^/D*, and the EQE reaches 32%, calculated using EQE = R(hc/qλ). A lower NEP signifies a higher sensitivity to weak optical signals. The extremely low NEP and picoampere‐scale dark current of our WSe_2_ device suggest that thermal noise dominates the overall noise spectrum.^[^ ^23^ ^]^ Under such conditions, thermal noise outweighs shot noise, explaining the excellent D* performance at zero bias observed in Figure 4e. In Figure S6 (Supporting Information), the device's responsivity and D* under 447 nm laser illumination were also examined. Both R and D* remain nearly constant over a wide range of optical power densities. Notably, under a forward bias of 1 V, D* increases by an order of magnitude to 1.07 × 10^12^ Jones, and the responsivity rises to 360 mA W^−1^. This improvement stems from the reduction in SBH under forward bias, which, despite slightly narrowing the depletion width, facilitates the collection of photogenerated carriers by making it easier for them to surmount the barrier. Although forward bias may increase dark current, the more pronounced increase in photocurrent can outweigh this, resulting in an overall enhancement of D*, especially in low‐noise device architectures. Since Figure 4e only presents data at zero bias, we can infer that even higher R and D* values can be expected under forward bias.

Figure S7 (Supporting Information) illustrates the photovoltaic performance of the device. Under zero bias, the short‐circuit current (I_sc_) refers to the photocurrent when *V_ds_* = 0 V, and the open‐circuit voltage (V_oc_) corresponds to the photovoltage when *I_ds_* = 0 A. From Figure 4b, V_oc_ and I_sc_ values under 633 nm illumination were extracted (Figure S7a, Supporting Information). The output electrical power (P_el_) reflects the device's ability to convert light into usable electrical energy and is calculated by P_el_ = V_ds_ × I_ds_, the maximum output power (P_max_) was derived (Figure S7b, Supporting Information). The fill factor (FF), indicating the quality of the photovoltaic output, is calculated using FF = P_max_ / (V_oc_ × I_sc_), and the power conversion efficiency (PCE) is defined as: PCE = P_max_ / (P_in_ × A), where P_in_ is the incident optical power and A is the effective area of the device. Under an illumination intensity of 497 mW cm^−2^, the P_max_ is shown in the shaded region of Figure S7c (Supporting Information).^[^ ^8b^ ^]^ As shown in Figure S7c (Supporting Information), the device achieves a P_max_ of 1.75 nW, a FF of 0.21, and a PCE of 0.8%. These values reflect moderate photovoltaic performance, with potential for further enhancement through optimization of contact interfaces, optical absorption, and depletion region engineering.^[^ ^24^ ^]^

Response time is a direct indicator of a photodetector's bandwidth and is one of its key performance metrics. The rise time (*τ_r_*) and fall time (*τ_f_*) are defined as the time it takes for the photocurrent to increase from 10% to 90% of its peak value, and decrease from 90% to 10%, respectively.^[^ ^25^ ^]^ The time‐resolved photoresponse behavior of the device was characterized under 532 nm laser illumination at a modulation frequency of 20 kHz in the absence of an external bias. As shown in Figure 4f, the device exhibits a *τ_r_* of 8.7 and a *τ_f_* of 3.8 µs. In contrast, under an applied bias of +1 V (Figure S8a, Supporting Information), the rise and fall times increased slightly to 29.2 µs and 4.2 µs, respectively. Under a reverse bias of –1 V (Figure S8b, Supporting Information), the rise and fall times were 42.1 µs and 12.5 µs, respectively. These results clearly demonstrate that the device exhibits a faster photoresponse under self‐powered operation compared to biased conditions. This improved response speed is attributed to the strong and fixed built‐in electric field present in the self‐powered state, which efficiently drives photogenerated carriers out of the depletion region. Figure S8c (Supporting Information) shows the frequency response of the WSe_2_ device, with a cutoff frequency reaching 80 KHz. The 3 dB bandwidth of our fabricated WSe_2_ Schottky diode reaches 80 KHz, which is consistent with the observed fast response times.^[^ ^8a^ ^]^

We also investigated the spectral response of devices with bottom and top electrodes under constant illumination intensity and varying bias voltages. Figure 4g displays the R as a function of incident wavelength ranging from 480 to 1000 nm. Notably, the spectral shape of R closely follows the absorption spectrum of the WSe_2_ thin film. A responsivity peak appears around 760 nm, which corresponds to the A exciton transition in bulk WSe_2_ (≈770 nm).^[^ ^26^ ^]^ The top‐contact Cr electrode device exhibits no photoresponse at zero bias and has a responsivity that is 200 times lower than that of the bottom‐contact device under a 1 V bias. The EQE comparison between the bottom contact device and the top contact one is also displayed in Figure S9 (Supporting Information). This disparity arises because the Cr bottom electrode does not block incoming light, and the device's top surface is either transparent, improving light coupling efficiency. Photogenerated carriers are created closer to the depletion region, enabling more effective carrier separation and improved photovoltaic performance. The bottom‐up architecture allows light to directly reach the active junction area and minimizes recombination losses, significantly enhancing the responsivity. In addition, the Schottky device also exhibits photoresponse in the near‐infrared region, such as at 900 and 1010 nm. As shown in Figure S10 (Supporting Information), we present the photocurrent responses at 900 and 1010 nm under varying optical power densities.

Figure 4h presents a spatially resolved scanning photocurrent microscopy (SPCM) image of the device obtained under 532 nm focused laser illumination at zero bias. During the measurement, the laser spot was scanned across the device surface, and the variation of the short‐circuit current with position was recorded. The photocurrent signal is primarily localized near the interface between the bottom Cr electrode and the WSe_2_ layer, indicating the presence of a pronounced Schottky barrier at this contact. Upon laser excitation, photogenerated carriers are predominantly generated in this interfacial region. Driven by the built‐in electric field, electrons and holes are efficiently separated and subsequently migrate toward the top electrode, resulting in the generation of a short‐circuit photocurrent.^[^ ^8a^ ^]^

As shown in Figure S11 (Supporting Information), the optoelectronic performance of the WSe_2_ device with a top electrode configuration was systematically characterized. The top‐electrode device exhibited a relatively low rectification ratio of only 232, with an ideality factor of 1.24. Based on its transient photoresponse under 447 nm illumination, the responsivity was determined to be 1.51 mA W^−1^, with a specific detectivity of 1.44 × 10^10^ Jones, and an external quantum efficiency (EQE) of 0.4%. Under 633 nm laser excitation, the rise and fall times were measured to be 39.9 µs and 11 µs, respectively. The cutoff frequency of the device was found to be 16.8 KHz. These results indicate that the overall photodetection performance of the top‐electrode device is significantly inferior to that of the BE‐Schottky PD, thereby confirming the substantial advantages offered by the bottom‐electrode architecture.

Figure 4i presents a performance comparison between our Cr/ WSe_2_/Au Schottky photodetector and various reported undoped Schottky photodetectors, in terms of both specific detectivity (D*) and response time (τ).^[^ ^27^ ^]^ The Cr/ WSe_2_/Au device developed in this work demonstrates an ultrahigh detectivity combined with fast response, outperforming most existing photodetectors. **Table** **1** provides a detailed comparison of the optical performance parameters between our device and other undoped Schottky photodetectors, highlighting the exceptional optoelectronic properties of the Cr/WSe_2_/Au Schottky photodetector.

**Table 1:** Comparison with intrinsic WSe_2_ Schottky photodetectors.

WSe_2_ photodetector	Operating Wavelength [nm]	Response Time[τr/τf]	Detectivity[Jones]	Self Powered	Refs.
Au‐WSe2(Pattern)‐Au	532	11ms/44ms	1.00E+10	Yes	[15a]
Au‐WSe2(2H)‐Au	534	37ms/110ms	1.50E+09	No	[27a]
Au‐WSe2(CVD)‐Au	532	20ms/30ms	1.11E+10	Yes	[27b]
Au‐WSe2‐Au(Bottom)	635	120ms/150ms	1.21E+10	No	[27c]
Gra‐WSe2‐Gra	532	100ms/100ms	3.00E+10	No	[27d]
Ag‐WSe2(PCB)‐Ag	670	680ms/1010ms	5.86E+10	No	[27e]
Gra‐WSe2‐Gra	532	42.9µs/56µs	4.48E+09	Yes	[13]
Au‐WSe2‐Gra	650	332µs/554µs	3.00E+12	Yes	[10]
TiN‐WSe2‐Ni	447	10µs/40µs	1.00E+11	Yes	[8b]
Cr(Bottom)‐WSe2‐(Se)Au	633	8.7µs/3.8µs	1.07E+12	Yes	this work

This key result is attributed to the synergistic optimization of the device structure and interface engineering, which together enable exceptional optoelectronic performance. First, the use of a bottom‐contact architecture effectively suppresses the dark current, leading to a significant enhancement in the specific detectivity. In addition, the bottom‐contact configuration forms an asymmetric Schottky barrier at the contact interface, which facilitates the establishment of a strong built‐in electric field even under zero external bias. This built‐in field enables the rapid and efficient separation of photo‐generated electron–hole pairs, minimizing recombination losses and thus enabling a fast photoresponse. To further improve the interface properties, a thermally evaporated Se buffer layer is introduced, playing a crucial role through physical shielding, chemical passivation, and electronic modulation. On one hand, the Se layer forms a van der Waals gap between the metal and WSe_2_, effectively isolating mechanical stress and chemical interactions, thereby mitigating the formation of interface defects. On the other hand, Se atoms passivate dangling bonds and selenium vacancies at the metal‐WSe_2_ interface, reducing trap‐assisted recombination. More importantly, the Se buffer also modulates the energy band alignment at the metal–semiconductor junction, alleviating Fermi‐level pinning and facilitating the formation of a strong and stable built‐in electric field. These combined mechanisms enable efficient carrier separation and collection even under zero bias, resulting in low‐noise, high‐sensitivity detection alongside a fast photoresponse.

## 3 Conclusion

In summary, we have demonstrated a high‐performance bottom‐embedded electrode Cr/ WSe_2_/Au Schottky photodetector, in which a Se buffer layer is employed to facilitate high‐quality ohmic contact. The device exhibits excellent optoelectronic performance, including high responsivity, ultra‐low noise, fast response speed, and outstanding specific detectivity (D*). Under 633 nm laser illumination, the responsivity remains stable and nearly constant across a wide range of incident light intensities. Under 447 nm illumination and a 1 V bias, the device achieves a responsivity of 360 mA W^−1^ and a detectivity as high as 1.07 × 10^12^ Jones, demonstrating exceptional sensitivity to weak light signals.

Moreover, the device exhibits remarkable temporal response characteristics. Under a ‐1 V reverse bias, the rise and fall times are 8.7 and 3.8 µs, respectively, corresponding to a 3 dB bandwidth of 80 KHz. Spectral response measurements reveal that the bottom electrode structure significantly enhances responsivity‐by two orders of magnitude compared to the top‐contact configuration under the same bias‐highlighting its advantages in improving light absorption and carrier collection efficiency.

The proposed device architecture successfully combines high responsivity, high sensitivity, and low power consumption. With its simplified structure, scalable fabrication process, and outstanding performance, this work offers an effective design strategy and practical pathway toward integrated platforms for future 2D optoelectronic devices.

## 4 Experimental Section

### Fabrication of WSe_2_ Schottky Photodetectors

The SiO_2_/Si substrates were first ultrasonically cleaned in acetone, ethanol, and deionized water for 5 min each to remove surface contaminants, followed by drying with a nitrogen gas stream. Device patterning was performed using a UV maskless photolithography system (TuoTuo Technology (Suzhou) Co., Ltd.). The substrates were then placed horizontally on the sample stage of a custom‐designed capacitively coupled electrode‐less plasma (CCEP) system. During plasma treatment, the RF generator was set to 300 W, with a working pressure maintained at 20 Pa. Nitrogen (N_2_) and sulfur hexafluoride (SF_6_) were introduced as precursor gases at flow rates of 7 and 15 sccm, respectively.

Bottom‐embedded Au/Cr electrodes (50 nm/5 nm) were deposited via electron beam evaporation and thermal evaporation. Few‐layer WSe_2_ flakes were mechanically exfoliated from bulk crystals using a polydimethylsiloxane (PDMS) stamping method and subsequently transferred onto the pre‐deposited Cr electrodes. Further device patterning was conducted using the UV maskless lithography system. Se/Au (5 nm/50 nm) electrodes were deposited via thermal evaporation. Finally, the device was annealed in a vacuum furnace at 230 °C for 6 h to complete the fabrication of the WSe_2_ Schottky photodetector. The device was stored at 24 °C in a shaded environment inside a sample box placed in a vacuum desiccator, without the introduction of inert gas or any additional encapsulation.

### Characterization and Optoelectronic Performance Testing

Atomic force microscopy (AFM) and Kelvin probe force microscopy (KPFM) measurements of the WSe_2_ thin films were performed using a Bruker Dimension Fast Scan system. Photoluminescence (PL) and Raman spectra were collected using a Renishaw in via Raman microscope with a 532 nm excitation laser. Electrical measurements of the WSe_2_ Schottky photodetectors under various low‐temperature conditions were carried out using a Keithley 4200 semiconductor parameter analyzer. The optoelectronic response of WSe_2_ Schottky photodetectors was tested under different laser wavelengths, both in ambient conditions and in dark/illuminated states, using a Keithley 2634B source meter.

## Conflict of Interest

The authors declare no conflict of interest.

## Supporting information



Supporting Information

## Data Availability

The data that support the findings of this study are available from the corresponding author upon reasonable request.

## References

[1] a) C. Xie , C. Mak , X. Tao , F. Yan , Adv. Funct. Mater. 2017, 27, 1603886;

[2] a) W. Li , Z. Chen , R. Yang , T. Mu , T. Yin , Y. Wang , Y. Wu , S. Wang , ACS Appl. Electron. Mater. 2023, 5, 5928;

[3] a) R. Singiri , D. W. Shin , B. Jo , P. Bathalavaram , M. Lee , M. G. Hahm , Y. L. Kim , ACS Appl. Electron. Mater. 2023, 5, 4778;

[4] a) L. Zhou , R. Qi , H. Nan , W. Wang , J. Bai , M. Wang , J. Jian , Z. Weng , Z. Cai , S. Xiao , X. Gu , J. Mater. Chem. C 2025, 13, 8274;

[5] a) C.‐H. Lee , G.‐H. Lee , A. M. van der Zande , W. Chen , Y. Li , M. Han , X. Cui , G. Arefe , C. Nuckolls , T. F. Heinz , J. Guo , J. Hone , P. Kim , Nat. Nanotechnol. 2014, 9, 676; 25108809 10.1038/nnano.2014.150

[6] C. Tan , H. Wang , X. Zhu , W. Gao , H. Li , J. Chen , G. Li , L. Chen , J. Xu , X. Hu , L. Li , T. Zhai , ACS Appl. Mater. Interfaces 2020, 12, 44934. 32909433 10.1021/acsami.0c11456

[7] a) T. Bu , X. Duan , C. Liu , W. Su , X. Hong , R. Hong , X. Zhou , Y. Liu , Z. Fan , X. Zou , L. Liao , X. Liu , Adv. Funct. Mater. 2023, 33, 2305490;

[8] a) Y. Zhang , K. Ma , C. Zhao , W. Hong , C. Nie , Z.‐J. Qiu , S. Wang , ACS Nano 2021, 15, 4405; 33587610 10.1021/acsnano.0c08075

[9] a) M. Dai , Q. Wu , C. Wang , X. Liu , X. Zhang , Z. Cai , L. Lin , X. Gu , K. Ostrikov , H. Nan , S. Xiao , Adv. Opt. Mater. 2024, 12, 2301900;

[10] C. Zhou , S. Zhang , Z. Lv , Z. Ma , C. Yu , Z. Feng , M. Chan , npj 2D Mater. Appl. 2020, 4, 46.

[11] a) Y. Liu , J. Guo , E. Zhu , L. Liao , S.‐J. Lee , M. Ding , I. Shakir , V. Gambin , Y. Huang , X. Duan , Nature 2018, 557, 696; 29769729 10.1038/s41586-018-0129-8

[12] G. Kwon , Y.‐H. Choi , H. Lee , H.‐S. Kim , J. Jeong , K. Jeong , M. Baik , H. Kwon , J. Ahn , E. Lee , M.‐H. Cho , Nat. Electron. 2022, 5, 241.

[13] L. Tong , C. Su , H. Li , X. Wang , W. Fan , Q. Wang , S. Kunsági‐Máté , H. Yan , S. Yin , ACS Appl. Mater. Interfaces 2023, 15, 57868. 38017658 10.1021/acsami.3c14331

[14] Y. Wang , X. Zhang , Micro machines 2024, 15, 761.

[15] a) F. Liu , Y. Yan , D. Miao , J. Xu , J. Shi , X. Gan , Y. Cheng , X. Luo , Appl. Surf. Sci. 2023, 616, 156444;

[16] X. Zhang , X. Duan , W. Niu , X. Liu , X. Zou , H. Huang , D. H. Mudiyanselage , H. Fu , B. Jiang , G. Liu , Z. Yang , IEEE Trans. Electron Devices 2022, 69, 5644.

[17] a) X. Zheng , A. Calò , E. Albisetti , X. Liu , A. S. M. Alharbi , G. Arefe , X. Liu , M. Spieser , W. J. Yoo , T. Taniguchi , K. Watanabe , C. Aruta , A. Ciarrocchi , A. Kis , B. S. Lee , M. Lipson , J. Hone , D. Shahrjerdi , E. Riedo , Nat. Electron. 2019, 2, 17;

[18] P.‐C. Shen , C. Su , Y. Lin , A.‐S. Chou , C.‐C. Cheng , J.‐H. Park , M.‐H. Chiu , A.‐Y. Lu , H.‐L. Tang , M. M. Tavakoli , G. Pitner , X. Ji , Z. Cai , N. Mao , J. Wang , V. Tung , J. Li , J. Bokor , A. Zettl , C.‐I. Wu , T. Palacios , L.‐J. Li , J. Kong , Nature 2021, 593, 211. 33981050 10.1038/s41586-021-03472-9

[19] a) L. Dou , Y. Yang , J. You , Z. Hong , W.‐H. Chang , G. Li , Y. Yang , Nat. Commun. 2014, 5, 5404; 25410021 10.1038/ncomms6404

[20] C. Bao , Z. Chen , Y. Fang , H. Wei , Y. Deng , X. Xiao , L. Li , J. Huang , Adv. Mater. 2017, 29, 1703209. 10.1002/adma.201703209 28846818

[21] Y. Tang , Z. Wang , P. Wang , F. Wu , Y. Wang , Y. Chen , H. Wang , M. Peng , C. Shan , Z. Zhu , S. Qin , W. Hu , Small 2019, 15, 1805545. 10.1002/smll.201805545 30786144

[22] a) Y. Fang , A. Armin , P. Meredith , J. Huang , Nat. Photonics 2019, 13, 1;

[23] X. Li , J. Chen , F. Yu , X. Chen , W. Lu , G. Li , Nano Lett. 2024, 24, 13255. 39320324 10.1021/acs.nanolett.4c03450

[24] a) W. Song , J. Chen , Z. Li , X. Fang , Adv. Mater. 2021, 33, 2101059; 10.1002/adma.202101059 34046946

[25] W. Zhang , M.‐H. Chiu , C.‐H. Chen , W. Chen , L.‐J. Li , A. T. S. Wee , ACS Nano 2014, 8, 8653. 25106792 10.1021/nn503521c

[26] Y. Zhang , J. Wang , B. Wang , J. Shao , J. Deng , C. Cong , L. Hu , P. Tian , R. Liu , S.‐L. Zhang , Z.‐J. Qiu , Adv. Opt. Mater. 2018, 6, 1800660.

[27] a) X. Zhang , Z. Ao , X. Lan , W. Li , Mater. Lett. 2024, 367, 136580;

