# Supplementary material for: Ultrafast Self‐Driven WSe2 Photodetectors with Bottom Schottky Contacts
Source: Adv Sci (Weinh). 2025 Aug 4;12(40):e10373. doi: 10.1002/advs.202510373 (PMC12561265; doi:10.1002/advs.202510373)
Supplement: Supplementary file 1 — Supporting Information [file ADVS-12-e10373-s001.doc]

Supporting Information

**Ultrafast self-driven WSe2 photodetectors with bottom Schottky contacts**

*Jian Li, Zhihao Wang, Jialing Jian, Zhengjin Weng, Qianqian Wu, Xingyu Zhou, Liangliang Lin, Xiaofeng Gu, Peng Xiao*, Haiyan Nan*, and Shaoqing Xiao**

**
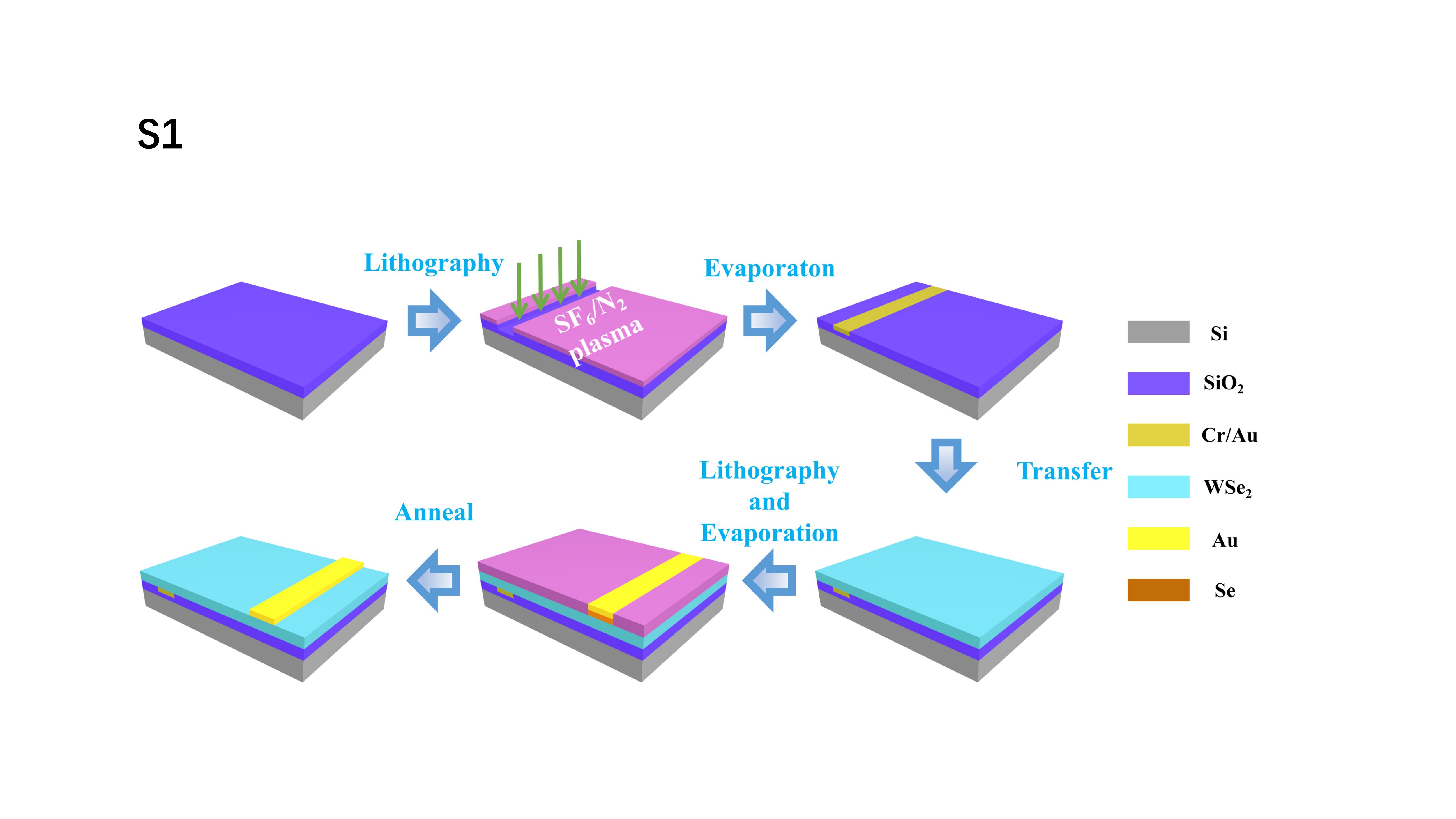
**

**Figure S1.** The fabrication process of the WSe_2_ Schottky photodetector is as follows: First, the SiO_2_/Si substrates were thoroughly cleaned to remove surface contaminants. After cleaning, photoresist was spin-coated onto the substrate, and the desired electrode pattern was defined using photolithography. The substrate was then placed horizontally on the sample stage of a custom-designed capacitively coupled electrode-less plasma system. Using nitrogen (N_2_) and sulfur hexafluoride (SF_6_) as precursor gases, 55 nm-deep grooves were etched into the surface. Au/Cr bottom electrodes (50 nm/5 nm) were deposited via a combination of electron-beam and thermal evaporation. Multi-layer WSe₂ flakes were mechanically exfoliated from bulk crystals using a polydimethylsiloxane (PDMS) stamping technique and transferred onto the pre-deposited Cr electrodes. A second round of spin coating and photolithography was used to define the top electrode pattern, followed by thermal evaporation of Se/Au electrodes (5 nm/50 nm). Finally, the device was annealed in a vacuum furnace at 230 °C for 6 hours, completing the fabrication of the WSe_2_ Schottky photodetector.^[1]^


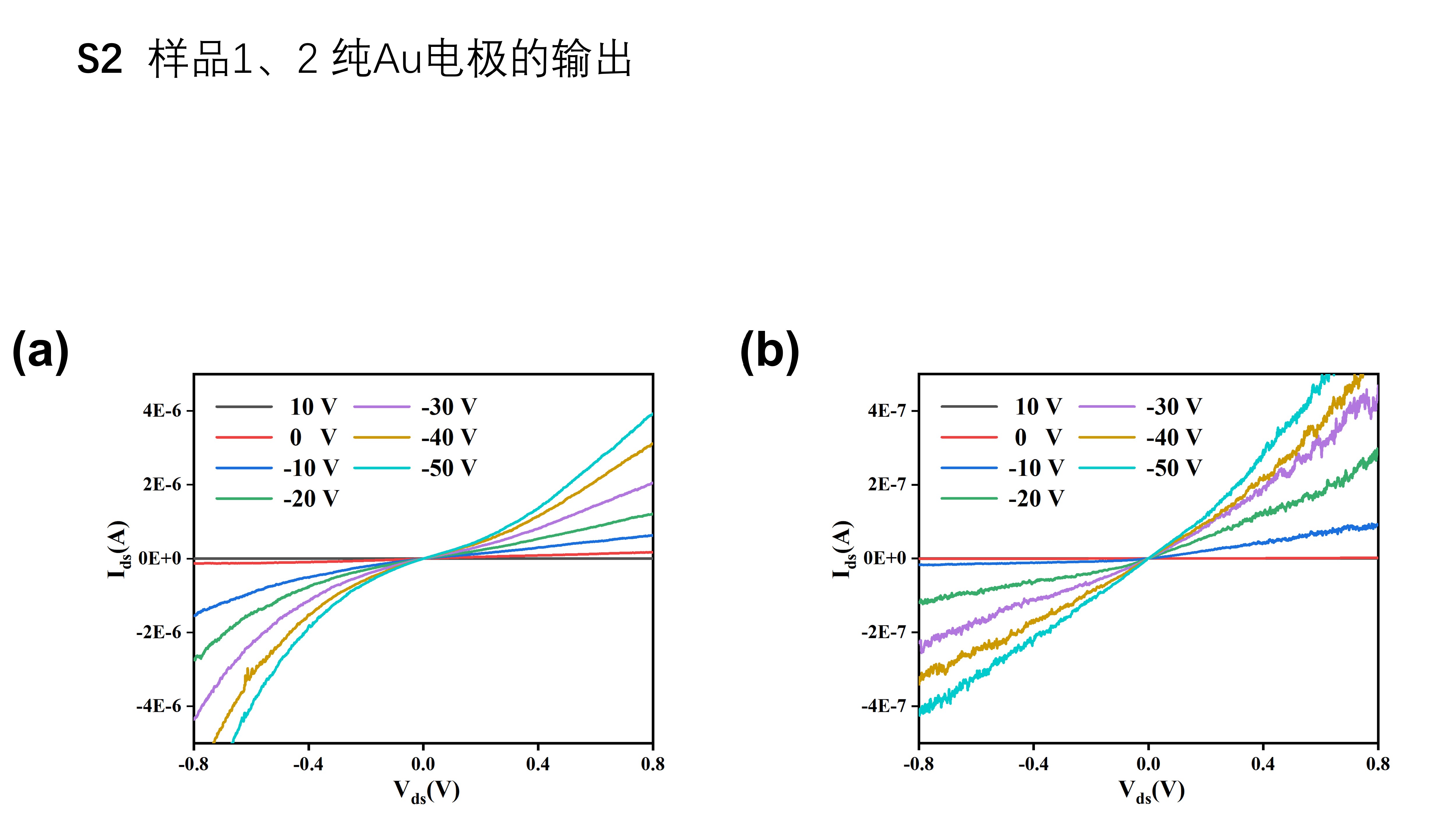


**Figures S2.** S2a and S2b show the output characteristic curves of two WSe_2_ devices with Au electrodes directly deposited on the top.


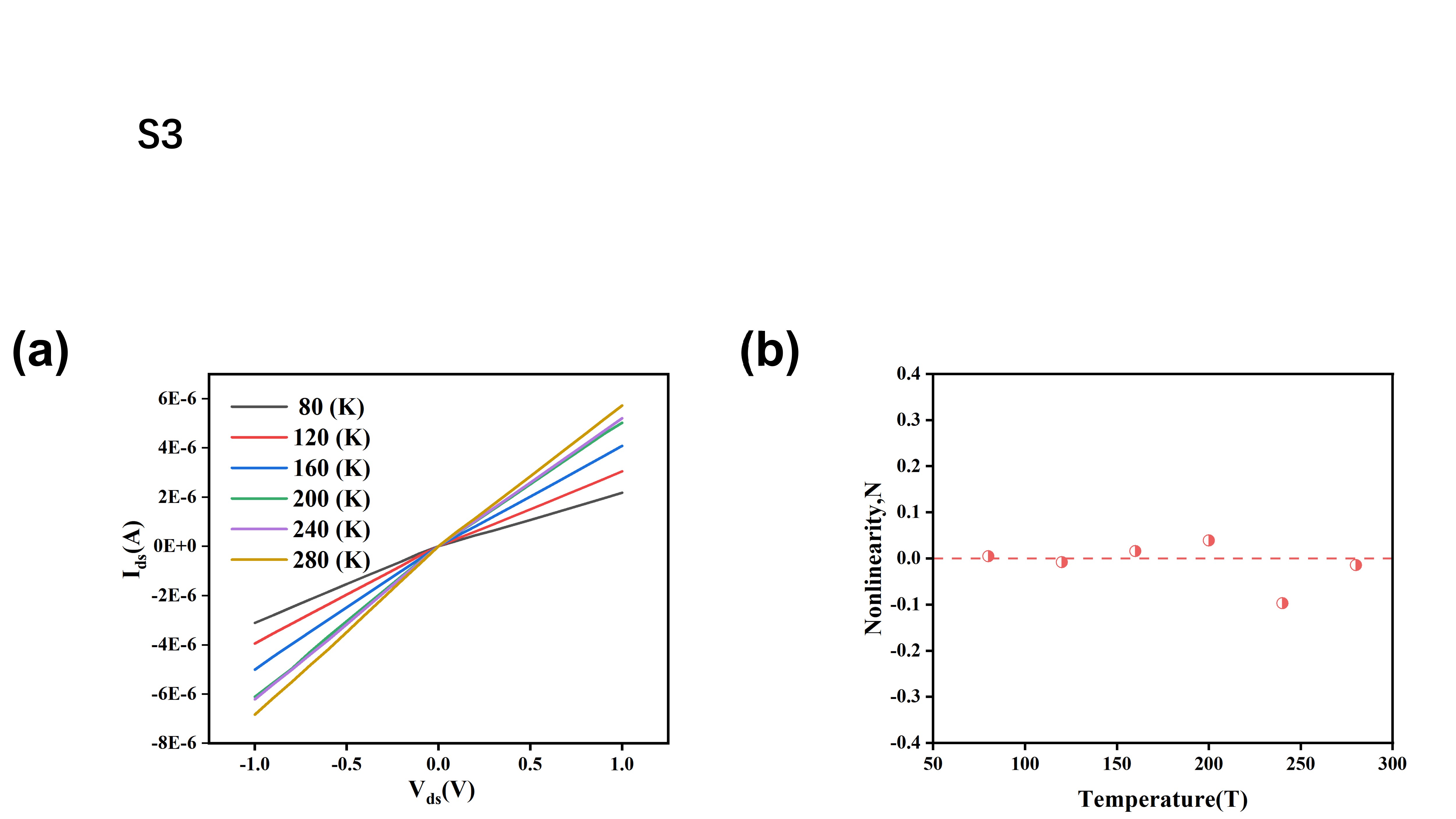


**Figure S3.** a) Output characteristics of the WSe_2_ transistor with Au-Au contacts at different temperatures under Vgs = -60 V. b) Nonlinear current-voltage characteristics of the WSe_2_ transistor with Au-Au contacts at different temperatures, extracted at V_ds_ = 1 V.


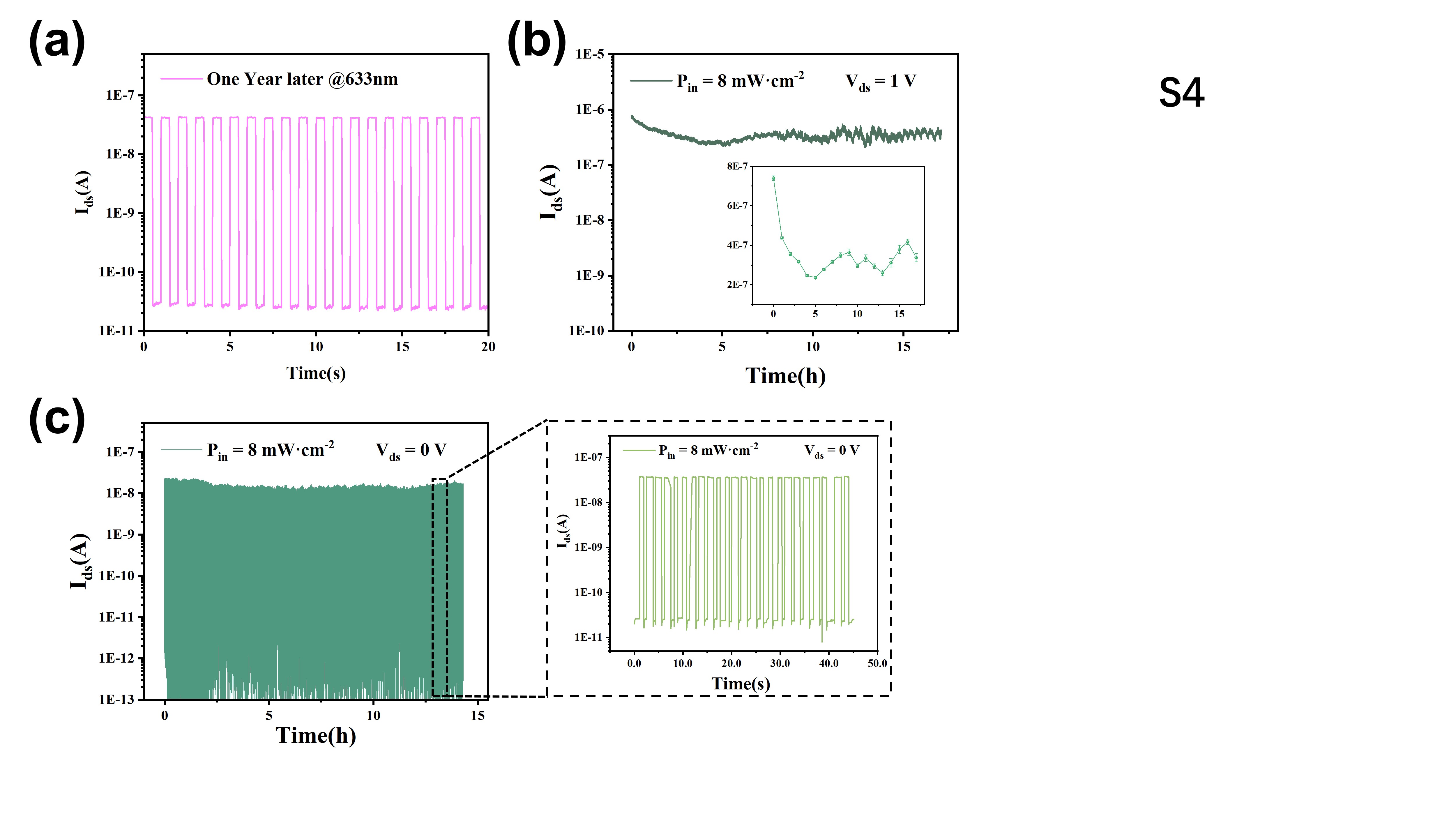


**Figure S4.** a) Time-resolved photoresponse measurement of the WSe_2_ device after one year of storage under 633 nm laser illumination. b) 17-hour continuous bias stability test. c) 14-hour on/off switching test under ambient conditions


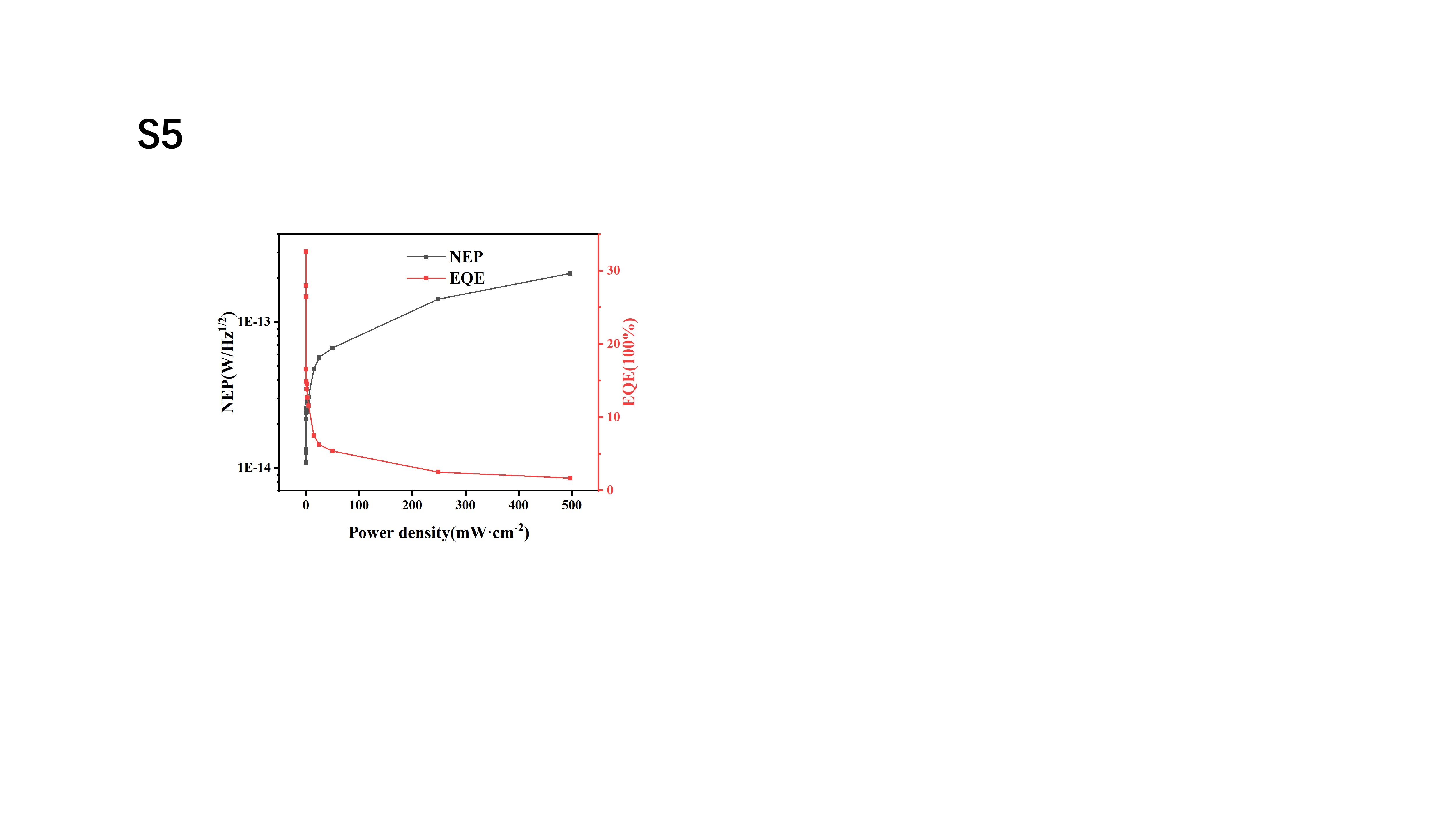


**Figure S5.** Noise equivalent power (NEP) and external quantum efficiency (EQE) of the WSe_2_ device under 633 nm laser illumination at zero bias, measured across a range of optical power densities from 0.05 to 497 mW·cm^-2^.


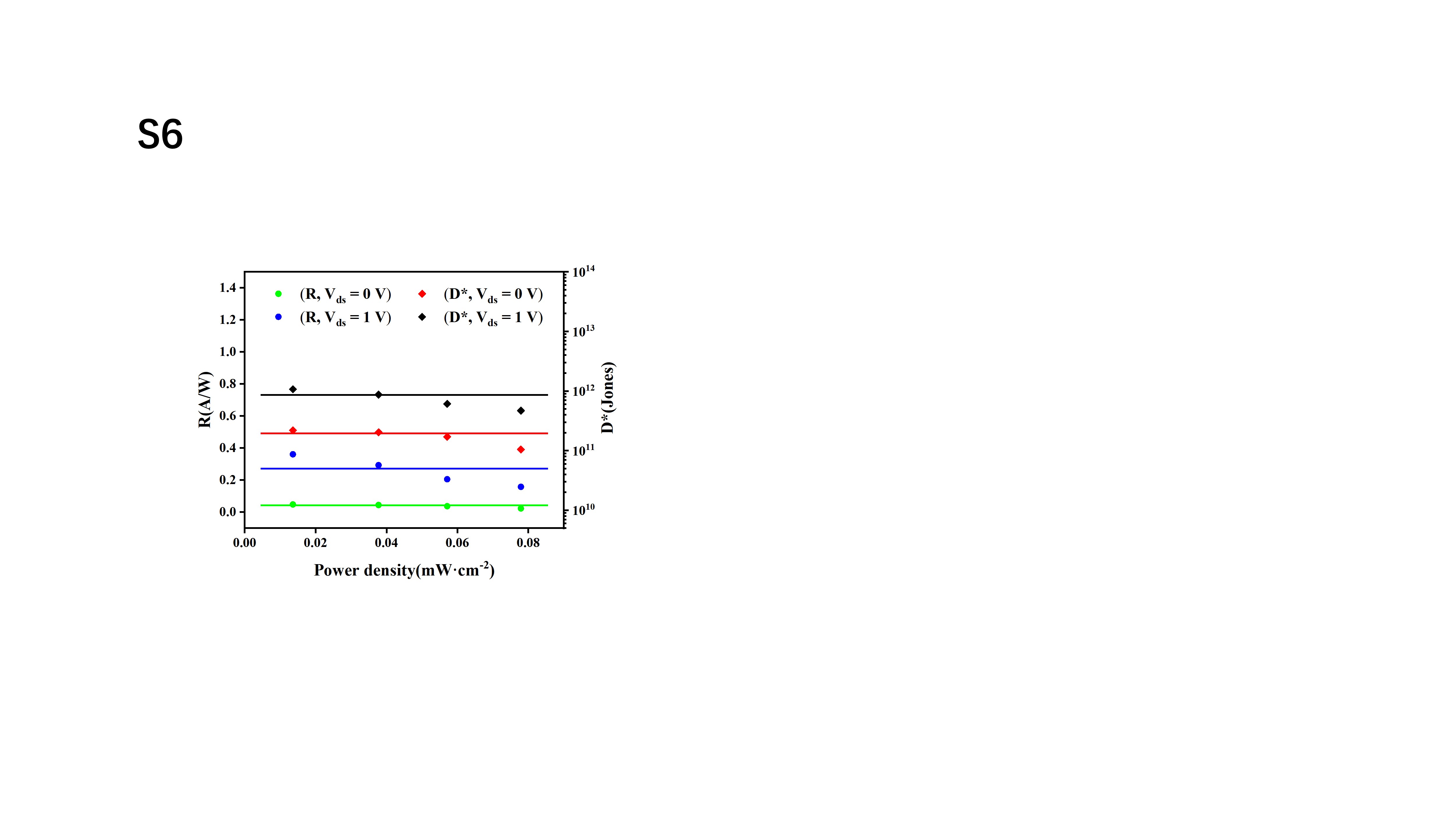


**Figure S6.** Responsivity (R) and detectivity (D*) of the WSe_2_ device under 447 nm laser illumination at various bias voltages.


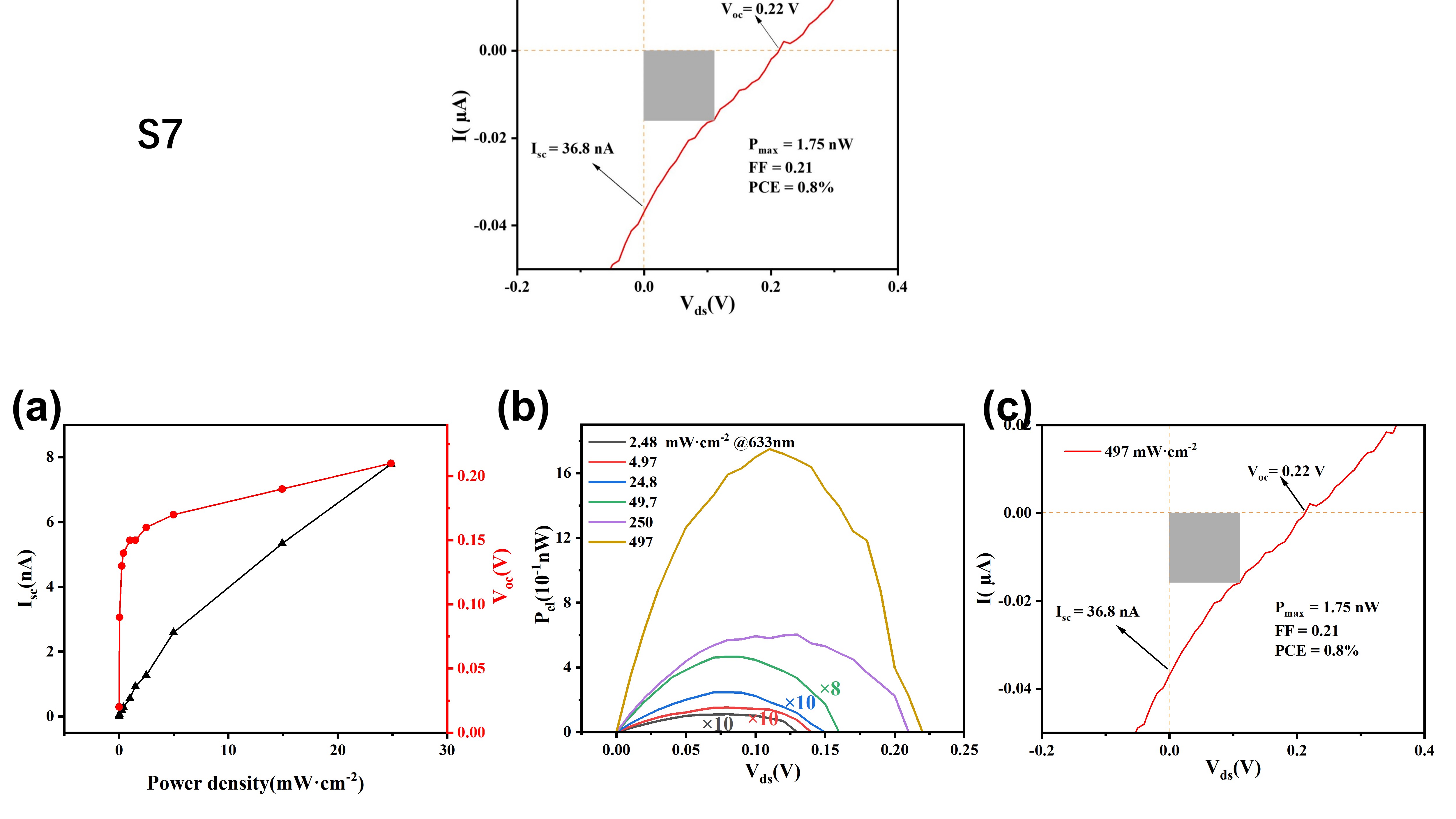


**Figure S7.** Photovoltaic response of the WSe_2_ Schottky photodetectors: a) Short-circuit current (I_SC_) and open-circuit voltage (V_OC_) as a function of optical power density. b) Output electrical power (P_el_) versus V_ds_. c) Output characteristics of the WSe_2_ Schottky photodetectors under light illumination.^[2]^


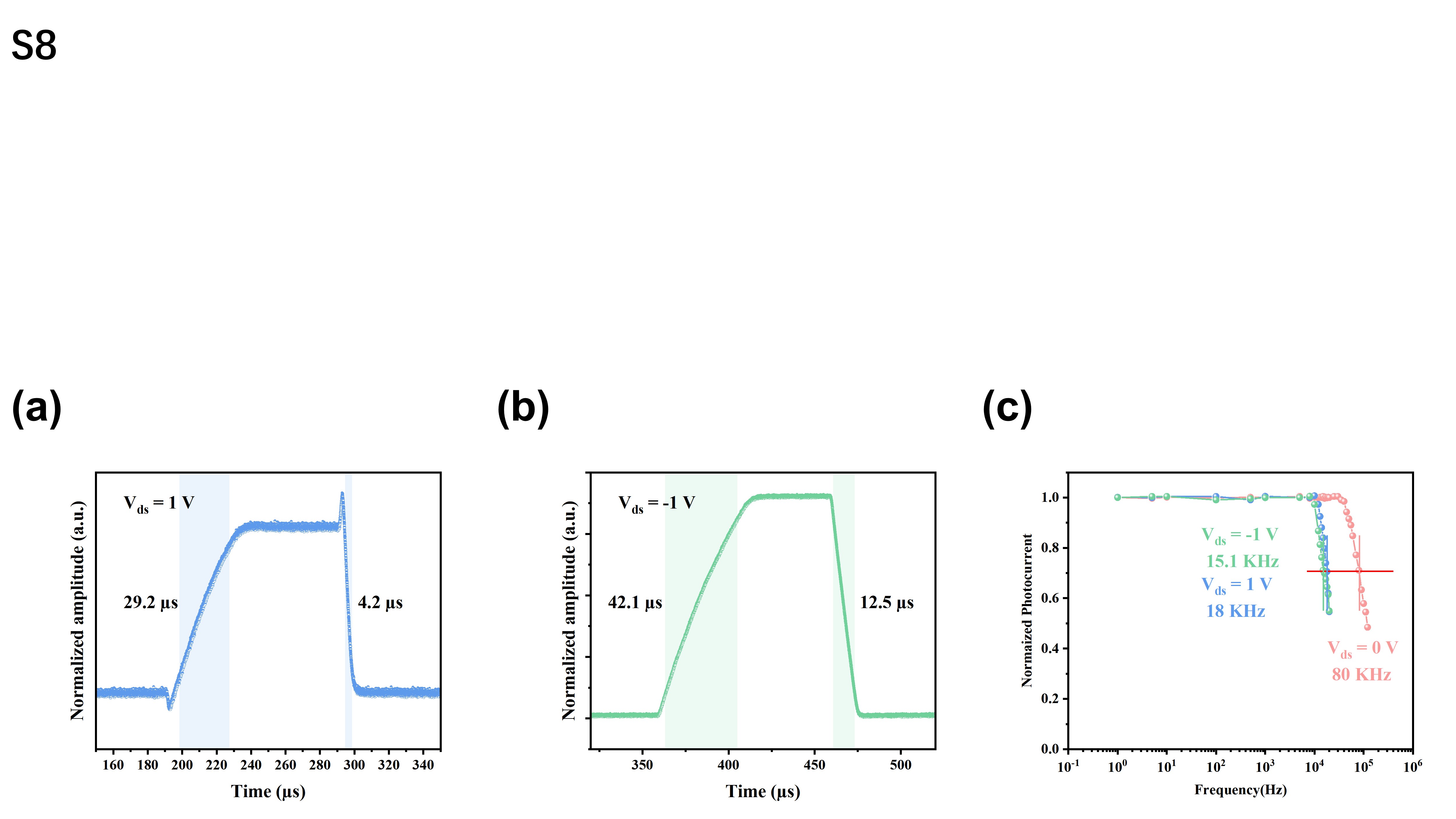


**Figure S8.** Response speed and cutoff frequency of the WSe_2_ device under different bias voltages: a) Response speed of the WSe_2_ device at a bias voltage of V_ds_ = 1 V. b) Response speed of the WSe_2_ device at a bias voltage of V_ds_ = –1 V. c) Cutoff frequency of the WSe_2_ device under varying bias voltages.^[3]^


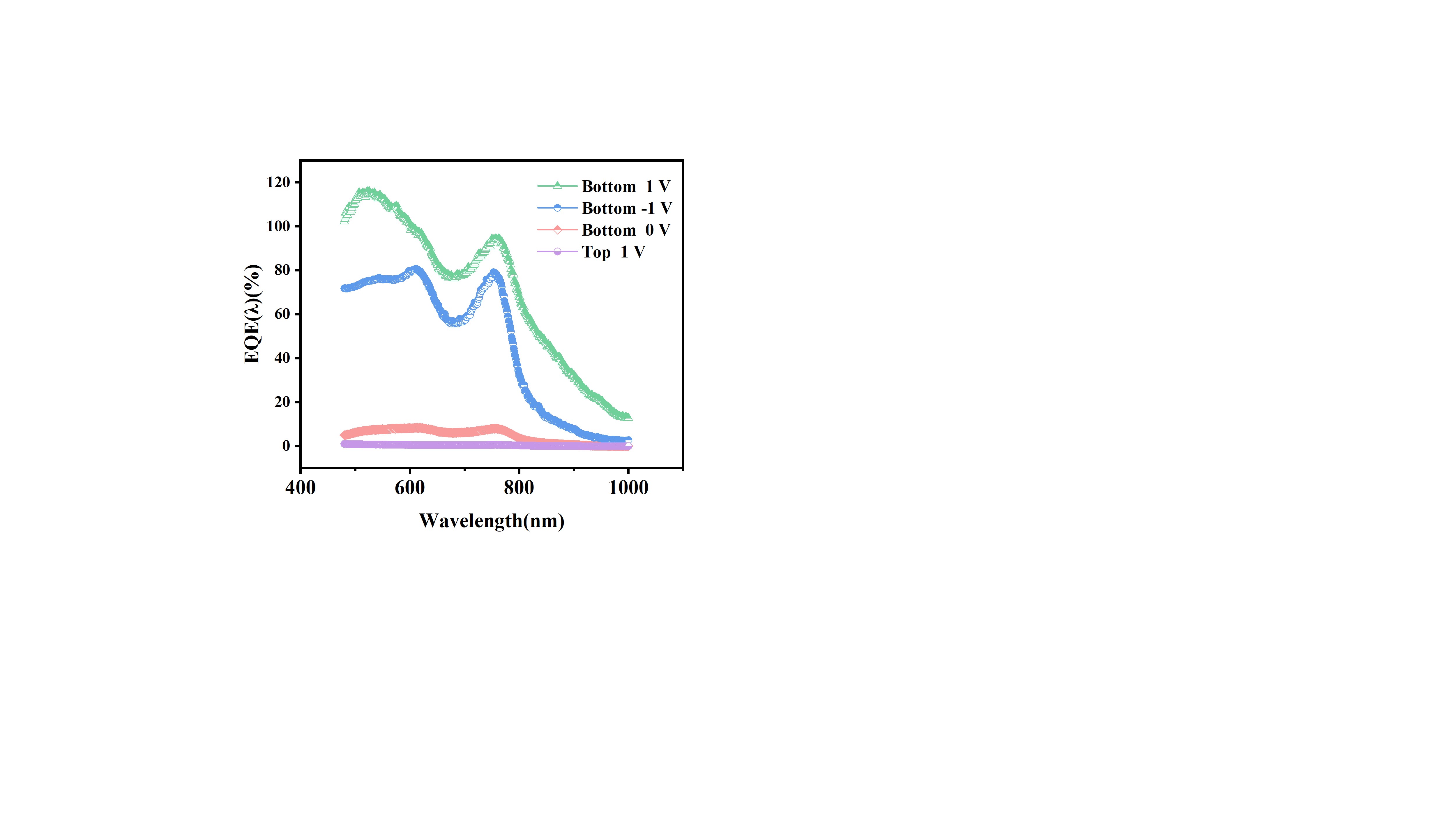


**Figure S9.** EQE of WSe_2_ devices with bottom and top electrodes under different V_ds_ values at constant optical power density.


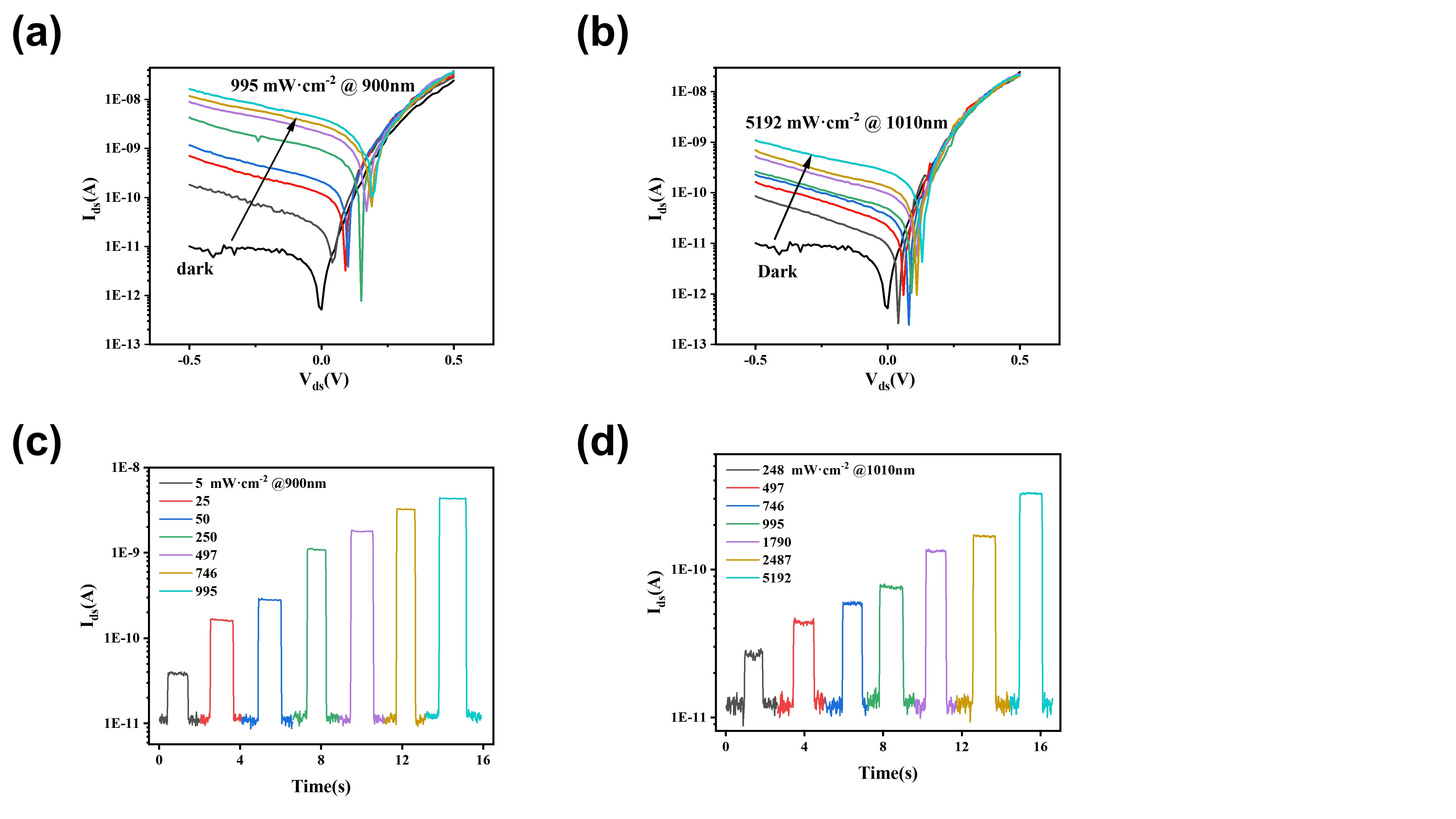


**Figure S10.** a, b) I_ds_-V_ds_ characteristics of the WSe_2_ Schottky diode under 900 nm and 1010 nm laser illumination. c, d) Photocurrent response under varying optical power densities.


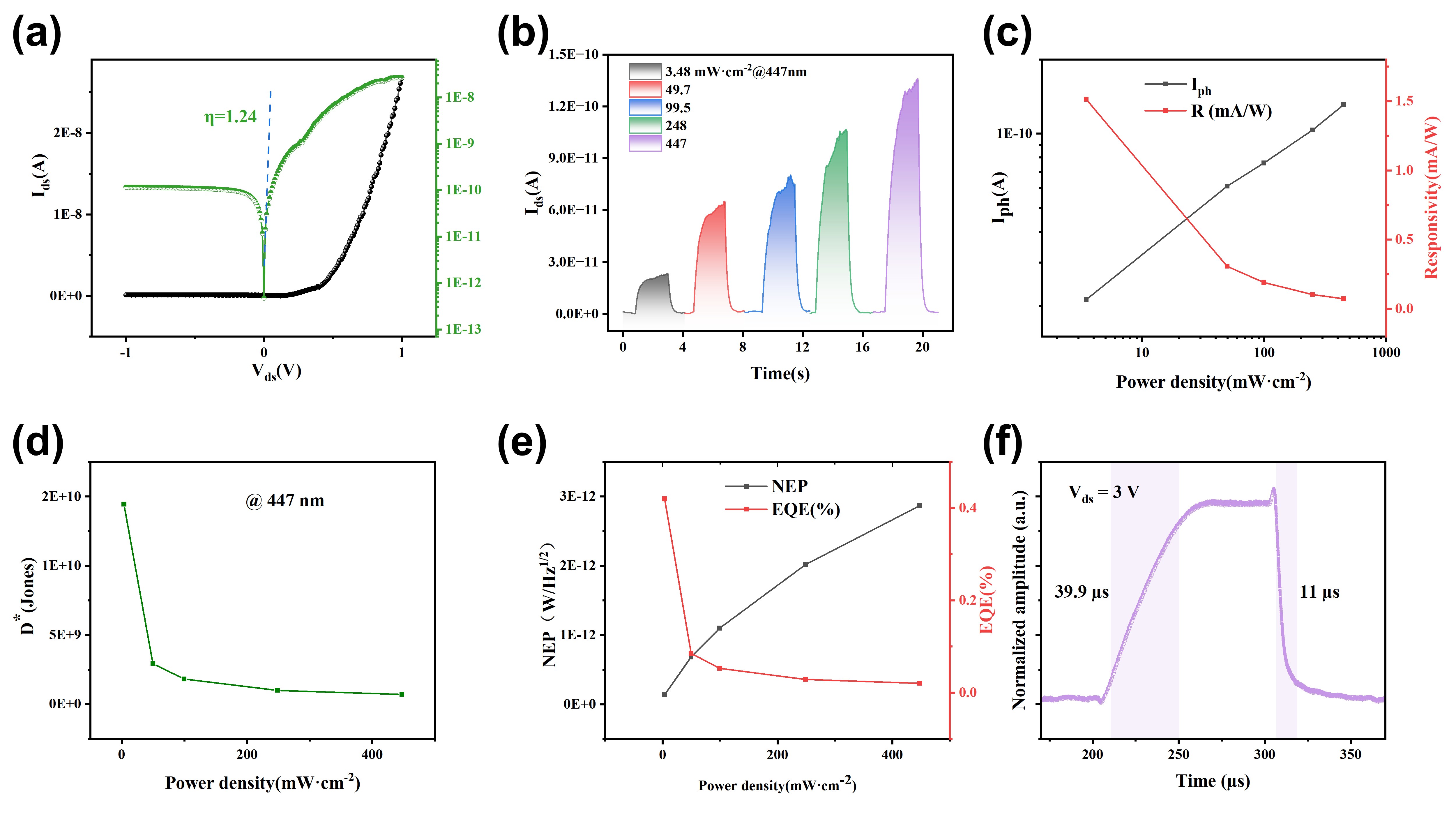


**Figure S11. Photodetection characteristics of the top-contact WSe_2_ Schottky** photodetectors**.** a) I_ds_-V_ds_ characteristics of the WSe**_2_** Schottky photodetectors, presented on both linear and logarithmic scales. b) Photocurrent response under 447 nm laser illumination at a bias voltage of 1 V, with incident optical power densities ranging from 3.48 to 447 mW·cm^-2^. c) Variation of photocurrent (I_ph_) and responsivity (R) with optical power density. d) Specific detectivity (D*) as a function of optical power density. e) Noise equivalent power (NEP) and external quantum efficiency (EQE) of the WSe**_2_** device under 447 nm laser illumination at V_ds_ = 1 V. f) Response speed of the WSe**_2_** device under a bias voltage of V_ds_ = 3 V.^[4]^

**References**

[1] L. Zhou, R. Qi, H. Nan, W. Wang, J. Bai, M. Wang, J. Jian, Z. Weng, Z. Cai, S. Xiao, X. Gu, *Journal of Materials Chemistry C* **2025**, 13, 8274.

[2] M. Zhang, X. Liu, X. Duan, S. Zhang, C. Liu, D. Wan, G. Li, Z. Xia, Z. Fan, L. Liao, *ACS Photonics* **2022**, 9, 132.

[3] Z. Wang, J. Jian, Z. Weng, Q. Wu, J. Li, X. Zhou, W. Kong, X. Xu, L. Lin, X. Gu, P. Xiao, H. Nan, S. Xiao, *Advanced Science* **2025**, n/a, 2417300.

[4] M. Dai, Q. Wu, C. Wang, X. Liu, X. Zhang, Z. Cai, L. Lin, X. Gu, K. Ostrikov, H. Nan, S. Xiao, *Advanced Optical Materials* **2024**, 12, 2301900.
